# Sympathetic nervous system activity and anti-lipolytic response to iv-glucose load in subcutaneous adipose tissue of obese and obese type 2 diabetic subjects

**DOI:** 10.1371/journal.pone.0173803

**Published:** 2017-03-27

**Authors:** Uwe Schumann, Christopher P. Jenkinson, Andreas Alt, Martina Zügel, Jürgen M. Steinacker, Marion Flechtner-Mors

**Affiliations:** 1 Division of Sports and Rehabilitation Medicine, Medical Center, University of Ulm, Ulm, Germany; 2 South Texas Diabetes and Obesity Institute, University of Texas Rio Grande Valley, Harlingen, Texas, United States of America; 3 Institute of Legal Medicine, University of Ulm, Ulm, Germany; Weill Cornell Medical College Qatar, QATAR

## Abstract

The study aim was to investigate the effect of endogenous insulin release on lipolysis in subcutaneous adipose tissue after adrenergic stimulation in obese subjects diagnosed with type 2 diabetes (T2D). In 14 obese female T2D subjects, or 14 obese non-T2D controls, glycerol concentration was measured in response to the α_1,2,_ß-agonist norepinephrine, the α_1_-agonist norfenefrine and the ß_2_-agonist terbutaline (each 10^−4^ M), using the microdialysis technique. After 60 minutes of stimulation, an intravenous glucose load (0.5 g/kg lean body mass) was given. Local blood flow was monitored by means of the ethanol technique. Norepinephrine and norfenefrine induced a four and three fold rise in glycerol dialysate concentration (p<0.001, each), with a similar pattern in adipose tissue. Following agonist stimulation and glucose infusion, endogenous insulin release inhibited lipolysis in the presence of norepinephrine, which was more rapid and pronounced in healthy obese controls than in T2D subjects (p = 0.024 obese vs T2D subjects). Insulin-induced inhibition of lipolysis in the presence of norfenefrine was similar in all study participants. In the presence of terbutaline the lipolysis rate increased two fold until the effect of endogenous insulin (p<0.001). A similar insulin-induced decrease in lipolysis was observed for each of the norfenefrine groups and the terbutaline groups, respectively. Adipose tissue blood flow remained unchanged after the iv-glucose load. Both norepinephrine and norfenefrine diminished blood flow slightly, but insulin reversed this response (p<0.001 over the entire time). Terbutaline alone and terbutaline plus increased endogenous insulin augmented local blood flow (p<0.001 over the entire time). In conclusion, a difference in insulin-induced inhibition of lipolysis was observed in obese T2D subjects compared to obese healthy controls following modulation of sympathetic nervous system activity and is assumed to be due to ß_1_-adrenoceptor mediated stimulation by norepinephrine.

## Introduction

Impaired adipose tissue metabolism in obesity plays a key role in obesity-related pathophysiology with diverse metabolic consequences, including hyperglycemia and insulin resistance [1,2]. Insulin promotes the uptake and intracellular metabolism of glucose in adipose tissue and inhibits lipolysis. Loss of insulin's anti-lipolytic action allows triglycerides in fat tissue to be broken down, freeing glycerol and non-esterified fatty acids (NEFA) [3]. Increased circulating NEFA concentrations associated with increased basal adipose tissue lipolysis contribute to peripheral insulin resistance [2,4].

The sympathetic nervous system (SNS) innervates white adipose tissue and its activation is necessary for lipolysis [5]. SNS activity has been reported to be either decreased, normal, or increased in obesity [6]. However, evidence has been accumulating that low sympathetic nervous system activity and reactivity and/or reduced sensitivity to sympathetic stimulation may play a role in the development and maintenance of obesity [7]. Along with insulin, catecholamines are the major hormonal regulators of adipose tissue metabolism acting via α_1_,_2_- and ß-adrenergic receptors [8–10]. In human adipose tissue, ß_1-3_-adrenoceptors increase lipolysis, and the ß_2_-adrenoceptor seems to be especially important for lipolysis in vivo, because of its high activity and concomitant strong stimulatory impact on adipocyte lipolysis and blood flow [11]. The ß_3_-adrenoceptor appears to be only a minor factor in the control of lipolysis [12]. With regard to the α-adrenoceptor an activation of lipolysis has been reported through α_1_-adrenoceptors [13,14], whereas inhibition is induced via α_2_-adrenoceptors [5]. Catecholamines, acting through ß-activation, inhibit insulin action in fat cells and thereby promote insulin resistance [15]. It has been further suggested that insulin-ß-adrenoceptor interactions also occur in adipocytes. Insulin can acutely reduce cell surface ß-adrenoceptor numbers in these cells, with a subsequent decrease in the lipolytic sensitivity to beta-adrenergic agonists [16].

Metabolic differences between non-T2D obese subjects, herein referred to as healthy, and T2D obese subjects are well established. Insulin resistance, with respect to anti-lipolysis, is present in T2D [17,18], but to a lesser extent in obesity alone [19]. Furthermore, catecholamine resistance to lipolysis due to impaired activity of ß_2_-adrenoceptors in subcutaneous adipocytes (in vitro) from upper-body-obese males, was more pronounced in T2D than in controls [20].

This study was designed to investigate the effect of endogenous insulin on catecholamine-stimulated lipolysis in the adipose tissue of obese T2D subjects compared to healthy obese controls using the well-established microdialysis technique [21].

## Patients and methods

### Subjects

**Fig 1:**
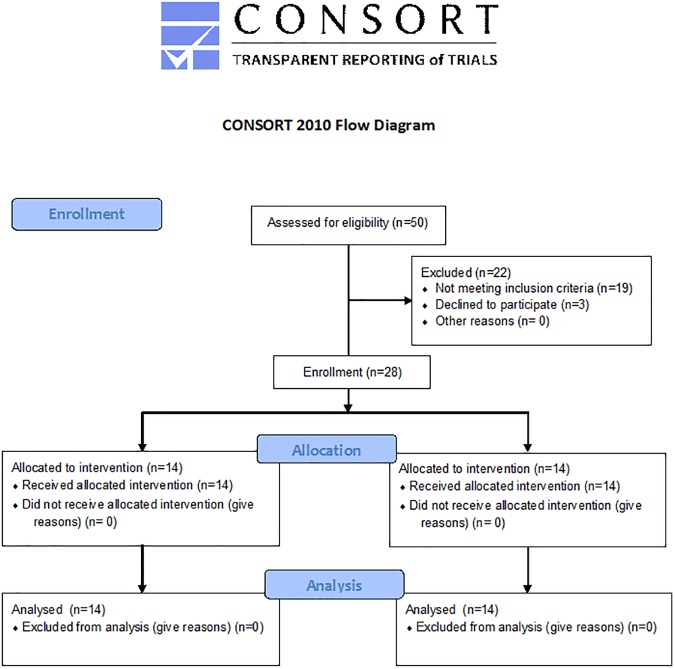
CONSORT flow diagram of patient recruitment.

Subjects were referred by their physicians to the University of Ulm Obesity Center for the treatment of obesity and type 2 diabetes. They were informed about the study and asked for voluntary participation. A flow chart of the 6-months patient recruitment is given in Fig 1. The experimental group was composed of 14 T2D female obese subjects. Study results were compared to 14 weight- and age-matched healthy female obese controls (control group). The number of subjects was considered to be sufficient for the detection of blood flow changes in adipose tissue [22]. The participants’ weight was stable for at least three months prior to the investigations. Clinical characteristics for both groups are given in Table 1. The diabetes duration ranged from 2 to 5 years. However, it should be noted that although patient’s diabetes has been diagnosed within these 5 years, an individual onset of the disease ahead of diagnosis cannot be excluded, conclusively early physiological changes with effects on sympathetic nervous system cannot be ruled out either.

**Table 1:** Clinical characteristics of female study subjects.

	T2D subjects (n = 14)	Controls (n = 14)
**Age (years)**	41.4±15.0	43.0±14.1
**Systolic blood pressure (mmHg)**	165.3±41.9	147.1±19.9
**Diastolic blood pressure (mmHg)**	94.6±16.9	91.4±12.4
**Body weight (kg)**	121.7±19.4	126.8±22.2
**Body mass index (kg/m**^**2**^**)**	45.2±7.6	44.7±6.0
**Percent body fat (%)**	45.0±5.6	41.8±6.5
**HbA1c (%)**	8.1±1.4	6.4±0.3*
**Blood glucose (mmol/L)**	6.9±2.3	5.2±0.6*
**Insulin (μU/mL)**	30.5±22.0	18.6±7.0
**Total cholesterol (mmol/L)**	5.7±0.9	5.4±0.9
**HDL-C (mmol/L)**	1.1±0.7	1.3±0.4
**Triglyceride (mmol/L)**	3.2±4.4	1.8±0.7

Values are means ± SD

*p< 0.05 type 2 diabetic subjects (T2D) vs. healthy obese controls

Six T2D subjects and five controls were on anti-hypertensive medication (diuretics, Ca-channel blockers). One week before the start of microdialysis experiments, all medication was discontinued. A registered dietitian advised participants to keep a balanced isoenergetic diet (20% of the energy derived from nutrients was from protein, 30% from fat, and 50% from carbohydrates) and to maintain their habitual exercise scheme. The investigation has been conducted according to the principles expressed in the Declaration of Helsinki. The study was approved by the Ulm University Ethics Committee and each participant gave written informed consent.

### Study protocol

On the day of the microdialysis investigations, participants were received at 8.00 a.m. in the research facility after an overnight fast and handled by the study nurse. Anthropometric measures were taken and thereafter subjects remained in the supine position for the remainder of the study. Body composition was measured by bioelectrical impedance analysis and blood pressure was determined by auscultation.

### Blood sampling procedure and glucose load

A teflon catheter was inserted into an ante-cubital vein for drawing blood samples. Venous blood was taken at baseline for clinical characteristics and every 30 minutes thereafter for the determination of glycerol, glucose, and insulin levels. Two hours after the start of the microdialysis experiments an intravenous (iv) glucose load was administered at a rate of 0.5 g glucose / kg lean body mass.

### Microdialysis experiments

For the microdialysis experiments, four catheters (30 x 0.3 mm Cuprophane, 3000 Da cut-off, glued onto 50 and 100 mm long sections of nylon tubing) were inserted into the abdominal subcutaneous adipose tissue of every subject without using anaesthesia, each 10 cm apart [21]. The catheters were connected to a high-precision pump (Perfusor VI, Braun, Melsungen, Germany) that delivered a flow rate of 2.5 μL/min and the catheters were perfused with saline buffer. For blood flow measurement, ethanol (100 mM) was added to the perfusion solutions [22]. No dialysate was collected 45 minutes after implantation. The experiments started at time 0 minutes.

One catheter was perfused with saline constantly (240 minutes) for basal glycerol values and for the assessment of insulin’s effect on lipolysis and local blood flow. The three other catheters were perfused with saline for 60 minutes and thereafter with either the α_1,2,_ß-agonist norepinephrine (10^−4^ M), the α_1_-agonist norfenefrine (10^−4^ M) or the ß_2_- agonist terbutaline (10^−4^ M). Two hours after the start of the experiment the iv-glucose load was given.

### Drugs and analytical methods

Norepinephrine was obtained from Hoechst Marion Roussel (Bad Soden, Germany), norfenefrine was purchased from Gödecke AG (Berlin, Germany) and terbutaline from Astra Zeneca GmbH (Wedel, Germany). Glycerol concentrations were analyzed with a bioluminescence method [23]. For ethanol measurements, two consecutive samples were combined. Ethanol concentrations were determined by gas-chromatography [24]. Glucose and insulin were measured by routine clinical methods.

### Statistics

Data are presented as the mean ± SEM unless otherwise stated. Differences in clinical characteristics with normal distributed parameters of both groups were determined by Student *t*-test. Differences between HbA1c and the serum lipid levels of each group were analyzed using the Mann-Whitney-U test. For paired comparison of not normal distributed values the Wilcoxon’s paired test was applied. Two-way ANOVA for repeated measurement was performed to determine between groups effects as well as within subject effects and group by time interactions. Time series data were collected for continuous variables, over time. For the comparison of intervention effects in subcutaneous adipose tissue between both groups at different times, time series of interest were determined with (i) basal (start of measurement to minute 60), (ii) adrenergic stimulation (60–120 minutes), and (iii) iv-glucose load (120–240 minutes). Single missing values were replaced with the mean of the observed values for that variable. Data for blood flow statistics were only partly available from one subject in both groups. A value of p<0.05 was considered significant. The software package SPSS version 21.0 (SPSS Inc., Chicago, IL) was applied for statistical analysis.

## Results

**Fig 2:**
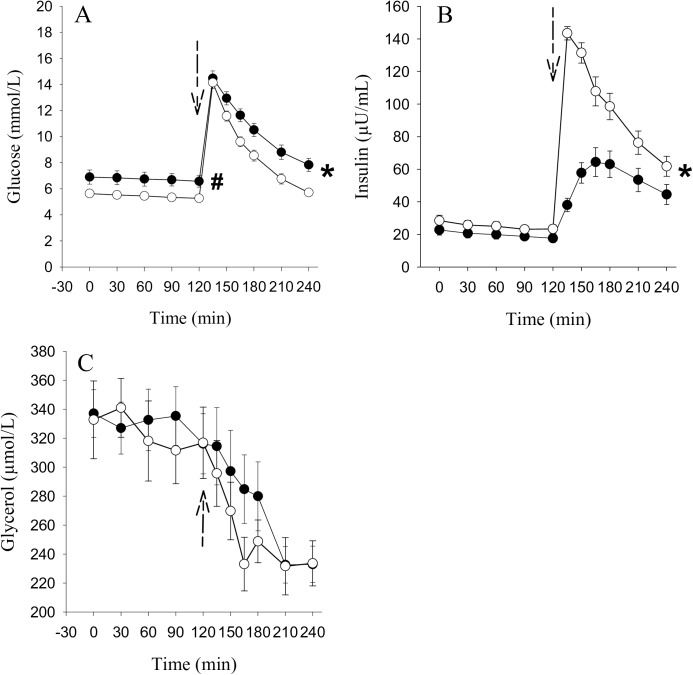
Glucose, insulin and glycerol concentrations in blood after an iv-glucose load. Glucose (Fig 2A), insulin (Fig 2B) and glycerol (Fig 2C) concentrations in venous plasma of 14 obese T2D subjects (●) and 14 obese controls (○). At time 120 minutes an iv-glucose load (0.5 g/kg lean body mass) was given (dotted arrow). Data are means ± SEM, ^#^p<0.05 time 0–120 minutes, *p<0.05 time 120–240 minutes obese T2D subjects vs. obese controls.

Fig 2 shows the changes of glucose, insulin, and glycerol concentration in the blood, when iv-glucose infusion was given 120 minutes after the start of the microdialysis experiment. Although the glucose concentration was higher in obese type 2 diabetic subjects under basal conditions (F = 7.1, p = 0.013) (Fig 2A), in both groups glucose concentrations rose rapidly to about 14 mM/L and then declined towards the baseline during the following 120 minutes. The reduction in glucose concentration was delayed in obese diabetic subjects compared to the controls (F = 6.6, p<0.001, interaction term). Basal insulin levels were similar in all patients; after the iv-glucose intervention, controls released significantly more endogenous insulin (F = 8.6, p<0.001, interaction term) (Fig 2B). Overall, in both groups insulin concentration returned towards the baseline, but was still elevated at the end of the study period. Plasma glycerol concentrations decreased similarly in both groups after glucose administration and remained stable at a low concentration during the last 30 minutes of the experiment (F = 13.9, p<0.001) (Fig 2C). Detailed statistical results are given in S1 File.

**Fig 3:**
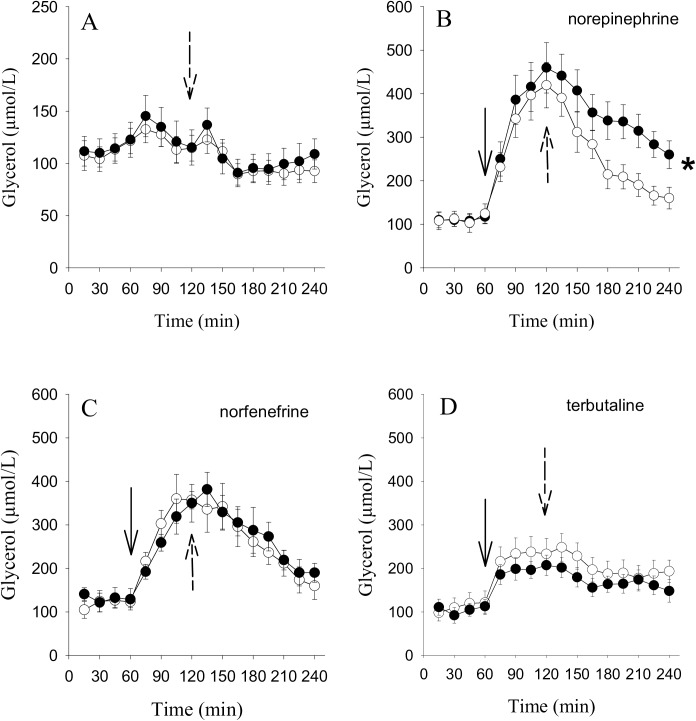
Glycerol microdialysate outflow in adipose tissue in response to adrenergic stimulation and after an iv-glucose load. Interstitial glycerol concentration in the abdominal subcutaneous adipose tissue of 14 obese T2D subjects (●) and 14 healthy obese controls (○). Fig 3A: Perfusion with saline during the duration of the experiment only. Fig 3B–3D: Perfusion with saline for 60 minutes. Then norepinephrine (Fig 3B), terbutaline (Fig 3C) or norfenefrine (Fig 3D), respectively, were added to the perfusate (solid arrow) at 10^−4^ M each. 120 minutes after time 0, an iv-glucose load was given intravenously (0.5 g/kg lean body mass) (dotted arrow). Data are means ± SEM, *p = 0.024 time 120–240 minutes obese T2D subjects vs. obese controls.

In the subcutaneous adipose tissue, the basal glycerol concentrations were stable until the interventions (Fig 3A–3D). The effect of the iv-glucose load on glycerol dialysate concentration, which is a significant reduction, is shown in Fig 3A with no group difference (F = 7.5, p<0.001).

In response to the α_1,2,_ß-agonist norepinephrine (Fig 3B), glycerol concentration increased over four-fold (F = 93.5, p<0.001). The administration of iv-glucose reversed this effect, the decline was faster in obese subjects, with a significant interaction between T2D subjects and controls (F = 2.9, p<0.001) and hence lower glycerol levels (F = 5.7, p = 0.024). In both groups glycerol dialysate concentration remained elevated compared to the baseline at the end of the experiment (p<0.001 T2D and p = 0.012 controls).

The α_1_-agonist norfenefrine induced a similar rise in glycerol compared with norepinephrine, although to slightly lower levels (Fig 3C). However, after the highest dialysate glycerol concentration was reached, the glycerol level declined to almost basal values after 240 min without a difference between the T2D and control subjects. The changes in glycerol concentration during the entire study period were significant (F = 65.3, p<0.001).

The ß_2_-adrenoceptor agonist terbutaline (Fig 3D) induced a significant two-fold increase of glycerol concentration and endogenous insulin decreased this outflow gradually. The change in glycerol levels was similar in all patients during the study period (F = 44.4, p<0.001).

Detailed statistical results for glycerol changes in adipose tissue in response to the interventions are given in S1 File.

The local blood flow in the tissue surrounding the microdialysis probe was investigated by measuring the escape of ethanol from the perfusion medium. For example, a decreased outflow to inflow ratio across the catheter indicates increased local blood flow.

**Fig 4:**
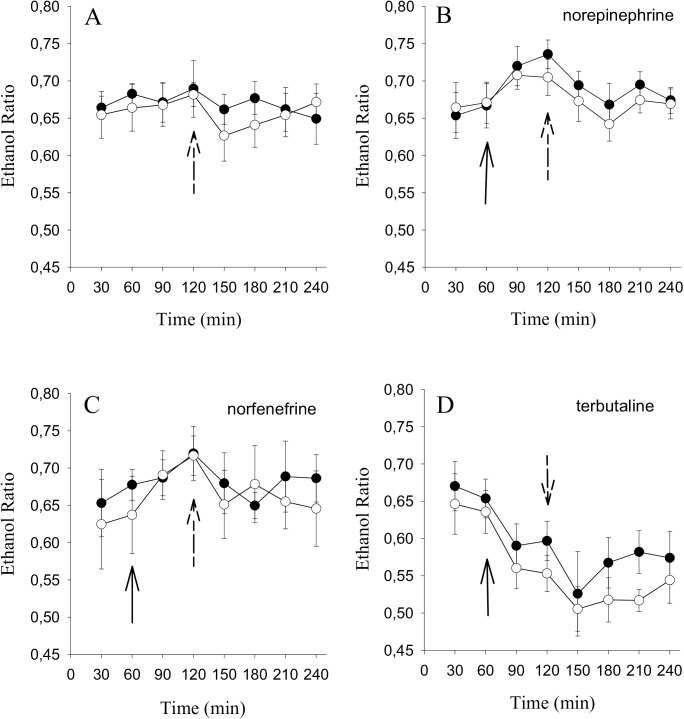
Change in adipose tissue blood flow in response to adrenergic stimulation and after an iv-glucose load. Ethanol ratio representing blood flow in the abdominal subcutaneous adipose tissue of 14 obese T2D subjects (•) and 14 obese controls (○). Fig 4A: Perfusion with saline during the duration of the experiment only. Perfusion with saline for 60 minutes and thereafter administration of norepinephrine (Fig 4B), norfenefrine (Fig 4C) or terbutaline (Fig 4D), respectively, at a concentration of 10^−4^ M each (solid arrow). 120 minutes after the start of the microdialysis experiments an iv-glucose load was given intravenously (0.5 g/kg lean body mass) (dotted arrow). Data are means ± SEM.

Fig 4A depicts the results of blood flow measurement under basal conditions and after the iv-glucose load. Basal blood flow remained unchanged (F = 0.6, p = 0.71) and the iv-glucose load induced only minor changes: notably, in the control group, a small acceleration occurred for 30 minutes in response to the glucose load. No such effect was observed in the diabetic patients.

Detailed statistical results for ethanol outflow changes in adipose tissue in response to the interventions are given in S1 File.

The effect of norepinephrine on local adipose tissue blood flow is depicted in Fig 4B. In both groups, the catecholamine induced a decrease in blood flow, whereas the iv-glucose load reversed this effect (F = 4.5, p<0.001).

The α_1_-agonist norfenefrine caused blood flow changes analogous to that observed after norepinephrine stimulation (Fig 4C). In all subjects local blood flow was inhibited initially, whereas the iv-glucose load triggered the opposite effect (F = 5.3, p<0.001).

The ß_2_-agonist terbutaline (Fig 4D) exerted a stronger effect on blood vessels. The agent alone, and even more so with the iv-glucose load, increased blood flow significantly (F = 12.4, p<0.0001).

## Discussion

In the present study, we addressed the question whether the lipolysis rate in subcutaneous adipose tissue of obese T2D subjects versus healthy obese controls is different after an iv-glucose load and hence the endogenous insulin release upon adrenergic stimulation.

The interstitial glycerol level in adipose tissue reflects lipolysis, since glycerol, in contrast to fatty acids, is metabolized by the tissue to an insignificant extent [25]. The iv-glucose load decreased glycerol concentration by approximately 30% in the blood of all subjects, and similarly in the subcutaneous adipose tissue, although in the healthy obese participants much more insulin was released. Insulin is generally considered to be the main factor in the inhibition of lipolysis, but obesity is a well-known risk factor for insulin resistance, with increased lipolysis, and development of T2D [26–28]. The higher insulin concentrations observed in the obese control subjects would be expected to result in a more pronounced inhibition of lipolysis. Fat cells respond differently to insulin, depending on factors such as their size, and a positive association has been shown between adipocyte size, fasting insulin levels and insulin resistance (HOMA-IR) [28–30]. The participants in our study were all severely obese and therefore different insulin concentrations might not be fully effective in these enlarged fat cells under basal conditions compared with adipocytes from lean healthy individuals. However, an interesting finding in this study is that although baseline glucose concentration in obese T2D was higher, after the iv-load peak glucose concentration was similar in both groups. The results suggest that T2D patients may have a better insulin sensitivity, not insulin resistance, at least at an acute glucose loading stimulation.

Norepinephrine perfusion induced a similar rise in adipose tissue glycerol dialysate concentration in all subjects. Catecholamine stimulation of lipolysis in nondiabetic and diabetic humans has been investigated previously, and consistent with our results the lipolysis rate was not different between the groups [31]. However, as we observed in this study, in response to the iv-glucose load the glycerol concentration declined, but the reduction was faster in the tissue of healthy obese subjects. This enhanced anti-lipolytic response in the obese healthy controls could be due to the higher insulin concentration after the glucose impact, which had a stronger effect on norepinephrine-stimulated lipolysis. In human adipose tissue catecholamines act on adipocytes via ß-adrenergic receptors, especially ß_1_- and ß_2_-receptors, and as reported, insulin powerfully inhibits this catecholamine-stimulated lipolysis [32]. Furthermore, fat cell lipolysis is activated via α_1_-adrenergic receptors [13,14]. Our results show that, in response to the α_1,2,_ß-norepinephrine and the α_1_-norfenefrine application, the glycerol concentration increased in a similar fashion. However, in the presence of norfenefrine, the reduction of the glycerol release induced by insulin was equal in all subjects. The results for the ß_2_-agonist terbutaline and the subsequent iv-glucose application show that the course of the glycerol dialysate concentration was consistently similar in both study groups. Overall, the combined findings suggest that insulin has different effect only via the ß_1_-adrenergic receptors, since after the stimulation of α_1_- and ß_2_-receptors no distinction was revealed.

The iv-glucose load and therefore insulin stimulation did not result in significant changes of adipose tissue blood flow (ATBF) in the subcutaneous adipose tissue of the study groups. It has been reported in previous studies that in lean individuals, blood flow accelerates after a glucose load, but in obesity and/or insulin-resistant subjects unaltered or lower ATBF and blunted postprandial responses have been observed [33–35]. Nevertheless, it has been suggested that insulin does not exert a direct effect on ATBF, rather insulin might be an important mediator, possibly acting via sympathetic activation or endothelial dysfunction [36,37]. A mutual interference between sympathetic nervous activity and hyperinsulinemia has been reported in lean individuals in previous investigations [38]. In our study the effect of insulin was determined after direct stimulation of adipose tissue with adrenergic agents to reveal the function of different adipocyte adrenoceptors. The stimulation of ß_2_-adrenoceptors explicitly increased blood flow alone and then was enhanced further, although temporarily, by insulin. Norepinephrine (and also norfenenfrine) first showed a decrease in blood flow and this effect was reversed by insulin. Beta-adrenoceptors are predominantly regarded as regulators and enhancers of adipose tissue blood flow [37,39]. This would mean that insulin overrides the effect of α_1_-adrenoceptors via the action of ß-receptors. However, under basal conditions blood flow did not change significantly in either group. Therefore, the insulin-induced effects in the presence of adrenergic agents cannot be explained by their stimulation and other mechanisms may be involved [37].

## Conclusions

In conclusion, the basal as well as adrenergic stimulated lipolysis rate in obese T2D subjects is not different from healthy obese controls. However, sympathetic stimulation of lipolysis and the anti-lipolytic response induced by endogenous released insulin are different and is merely assumed to be caused by ß_1_-adrenoceptors. Blood flow changes show the influence of insulin upon sympathetic stimulation with acceleration, but the underlying mechanisms remains to be elucidated.

## Supporting information

S1 File Results of ANOVA for repeated measurement statistics. (DOCX)

S2 File Trend statement checklist. (PDF)
