# Supplementary material for: Sympathetic nervous system activity and anti-lipolytic response to iv-glucose load in subcutaneous adipose tissue of obese and obese type 2 diabetic subjects
Source: PLoS One. 2017 Mar 27;12(3):e0173803. doi: 10.1371/journal.pone.0173803 (PMC5367786; doi:10.1371/journal.pone.0173803)
Supplement: S1 File — (DOCX) [file pone.0173803.s001.docx]

**S1 File. Results of ANOVA for repeated measurement statistics.**

| **Metabolite** | **Time (min)** | | | | | |
| --- | --- | --- | --- | --- | --- | --- |
|  | 0-240 | | 0-120 | | 120-240 | |
|  | F | p | F | p | F | p |
| Glucose (mmol/L) |  |  |  |  |  |  |
| within subjects effect | 475.5 | <0.001 | 2.7 | <0.05 | 475.6 | <0.001 |
| between group effect | 8.0 | <0.01 | 7.1 | <0.05 | 7.7 | <0.05 |
| interaction | 4.2 | <0.001 |  | ns | 6.6 | <0.001 |
| Insulin (µU/mL) |  |  |  |  |  |  |
| within subjects effect | 28.5 | <0.001 | 5.4 | <0.01 | 20.5 | <0.001 |
| between group effect |  | ns |  | ns | 4.3 | <0.05 |
| interaction | 7.3 | <0.001 |  | ns | 8.6 | <0.001 |
| Glycerol (µmol/L) |  |  |  |  |  |  |
| within subjects effect | 19.9 | <0.001 |  | ns | 13.9 | <0.001 |
| between group effect |  | ns |  | ns |  | ns |
| interaction |  | ns |  | ns |  | ns |

**Table A. Changes in blood parameters.** Differences in glucose, insulin and glycerol concentrations in blood during fasting conditions for 120 min and after an iv-glucose load (0.5 g glucose/kg lean body mass) at time 120 min in 14 obese subjects with type 2 diabetes and 14 obese non-T2D controls are shown.

| **Intervention** | **Time (min)** | | | | | |
| --- | --- | --- | --- | --- | --- | --- |
|  | 0-240 | | 0-120 | | 120-240 | |
|  | F | p | F | p | F | p |
| Norepinephrine |  |  |  |  |  |  |
| within subjects effect | 58.3 | <0.001 | 93.5 | <0.001 | 36.4 | <0.001 |
| between group effect |  | ns |  | ns | 5.7 | <0.05 |
| interaction | 2.9 | <0.001 |  | ns |  | ns |
| Norfenefrine |  |  |  |  |  |  |
| within subjects effect | 65.3 | <0.001 | 134.1 | <0.001 | 40.9 | <0.001 |
| between group effect |  | ns |  | ns |  | ns |
| interaction |  | ns |  | ns |  | ns |
| Terbutaline |  |  |  |  |  |  |
| within subjects effect | 44.4 | <0.001 | 82.8 | <0.001 | 11.5 | <0.001 |
| between group effect |  | ns |  | ns |  | ns |
| interaction |  | ns |  | ns |  | ns |

**Table B. Changes in glycerol concentrations in abdominal subcutaneous adipose tissue after interventions.** Fourteen obese subjects with type 2 diabetes and 14 obese non-T2D controls were investigated under basal conditions for 60 min. At time 60 min perfusion of the adipose tissue with the α_1,2,_ß-agonist norepinephrine, the α_1_-agonist norfenefrine or the ß_2_-agonist terbutaline (each 10^-4^ M) was allowed. At time 120 min, an iv-glucose load (0.5 g glucose/kg lean body mass) was applied.

| **Intervention** | **Time (min)** | | | | | |
| --- | --- | --- | --- | --- | --- | --- |
|  | 0-240 | | 0-120 | | 120-240 | |
|  | F | p | F | p | F | p |
| Norepinephrine |  |  |  |  |  |  |
| within subjects effect | 4.4 | <0.001 | 7.6 | <0.001 | 5.3 | <0.01 |
| between group effect |  | ns |  | ns |  | ns |
| interaction |  | ns |  | ns |  | ns |
| Norfenefrine |  |  |  |  |  |  |
| within subjects effect | 16.4 | <0.001 | 16.4 | <0.001 | 5.7 | <0.001 |
| between group effect |  | ns |  | ns |  | ns |
| interaction |  | ns |  | ns |  | ns |
| Terbutaline |  |  |  |  |  |  |
| within subjects effect | 12.4 | <0.001 | 6.7 | <0.001 | 2.7 | <0.05 |
| between group effect |  | ns |  | ns |  | ns |
| interaction |  | ns |  | ns |  | ns |

**Table C. Changes in adipose tissue blood flow in abdominal subcutaneous adipose tissue after interventions.** Fourteen obese subjects with type 2 diabetes and 14 obese non-T2D controls were investigated under basal conditions for 60 min. At time 60 min perfusion of the adipose tissue with the α_1,2,_ß-agonist norepinephrine, the α_1_-agonist norfenefrine or the ß_2_-agonist terbutaline (each 10^-4^ M) was allowed. At time 120 min, an iv-glucose load (0.5 g glucose/kg lean body mass) was applied.
